# Smooth Muscle Tumor of Uncertain Malignant Potential (STUMP) in the Inguinal Soft Tissue: A Rare Case Requiring Differentiation From Metastatic Lymphadenopathy

**DOI:** 10.7759/cureus.78642

**Published:** 2025-02-06

**Authors:** Yuki Ando, Risako Ito, Hiromi Matsuo, Kyoko Baba

**Affiliations:** 1 Plastic Surgery, Kitasato University Medical Center, Kitamoto, JPN; 2 Department of Plastic and Aesthetic Surgery, Kitasato University School of Medicine, Sagamihara Kanagawa, JPN

**Keywords:** groin, leiomyoma with bizarre nuclei, metastatic lymphadenopathy, smooth muscle tumor, smooth muscle tumor of uncertain malignant potential, soft-tissue tumor

## Abstract

We report a rare case of a smooth muscle tumor of uncertain malignant potential arising in the inguinal soft tissue, requiring differential diagnosis from metastatic lymphadenopathy. The patient was a 74-year-old male.

On the initial examination, a painless, elastic-firm mass measuring approximately 25 × 15 mm was palpated slightly cephalad and medial to the left inguinal region. The mass was non-adherent to the overlying skin or underlying tissue.

Based on preoperative imaging findings, metastatic lymphadenopathy was considered likely. However, further evaluation by the urology department found no definitive evidence of prostate cancer, and cystoscopy ruled out bladder cancer. Under local anesthesia, the tumor was excised and subjected to histopathological analysis.

Histopathological examination with hematoxylin and eosin staining revealed a fibrous capsule surrounding the tumor and spindle-shaped cells with atypical nuclei arranged in loose bundles and fascicles within the tumor. Immunohistochemical staining showed positive results for desmin and alpha smooth muscle actin, while S-100 protein and CD34 were negative. B cell lymphoma 2 and Signal transducer and activator of transcription 6 were weakly positive, while β-catenin was negative. Thus, the histopathological findings were assessed, and the tumor was classified as a smooth muscle tumor of uncertain malignant potential.

The patient’s postoperative course was uneventful, with no evidence of recurrence at two years post-surgery.

Smooth muscle tumor of uncertain malignant potential is defined as a “soft tissue tumor of uncertain malignant potential that does not meet the diagnostic criteria for conventional leiomyoma, its various subtypes, or leiomyosarcoma.” To our knowledge, this case represents an exceedingly rare occurrence of smooth muscle tumor of uncertain malignant potential in the field of plastic and orthopedic surgery.

## Introduction

Soft tissue tumors are a group of non-epithelial tumors originating from soft tissues such as subcutaneous tissue, muscle, nerves, and blood vessels, or parenchymal organs [1]. These tumors can occur in any part of the body and encompass a wide variety of diseases. According to the 2020 revision of the World Health Organization (WHO) Classification of Bone and Soft Tissue Tumors, bone tumors are categorized into 51 types, while soft tissue tumors are classified into 135 types [1]. The diagnosis of soft tissue tumors is based on this classification, which also guides treatment planning [1,2]. 

Smooth muscle tumors (STs) are neoplasms of smooth muscle cells and are included in the WHO Classification of Bone and Soft Tissue Tumors [1-3]. While STs commonly originate in the uterus, those occurring in soft tissues are typically found in the retroperitoneum or abdominopelvic cavity [1]. Consequently, STs are well-recognized in gynecology [4]. In contrast, in plastic surgery, benign STs classified as leiomyomas (LMs) are considered rare skin tumors [5-7]. Multiple LMs of the skin are also known to be a manifestation of Reed syndrome [5,7]. However, STs seldom occur in the soft tissues treated under the scope of plastic and orthopedic surgery. Based on a search conducted on PubMed in December 2024, we were only able to identify three reported cases of soft tissue LMs of the extremities in these fields [8-10]. While reports on angioleiomyomas exist [11,12], the 2013 WHO Classification of Bone and Soft Tissue Tumors separated angioleiomyomas from STs, establishing the latter as an independent category [13].

We encountered a rare case of an ST originating in the soft tissue of the inguinal region. Preoperatively, clinical findings, medical history, and imaging findings strongly suggested malignant lymph node metastasis. However, histopathological examination led to the diagnosis of LM with bizarre nuclei. Following the revision of the WHO Classification of Bone and Soft Tissue Tumors to its 5th edition [1], which was completed soon after surgery, the diagnosis was updated to smooth muscle tumor of uncertain malignant potential (STUMP). STUMP is defined as an ST of uncertain malignant potential that does not meet the diagnostic criteria for typical LM or leiomyosarcoma. The latest (5th) edition of the WHO Classification introduced STUMP under the ST category and placed STUMP in the intermediate-grade tumor category [3]. STUMP in the fields of plastic and orthopedic surgery is rarer. To our knowledge, based on a literature search conducted in December 2024 using PubMed, no reports similar to our case have been identified. We report this rare case along with some insights gained through our clinical experience.

## Case presentation

**Figure 1:**
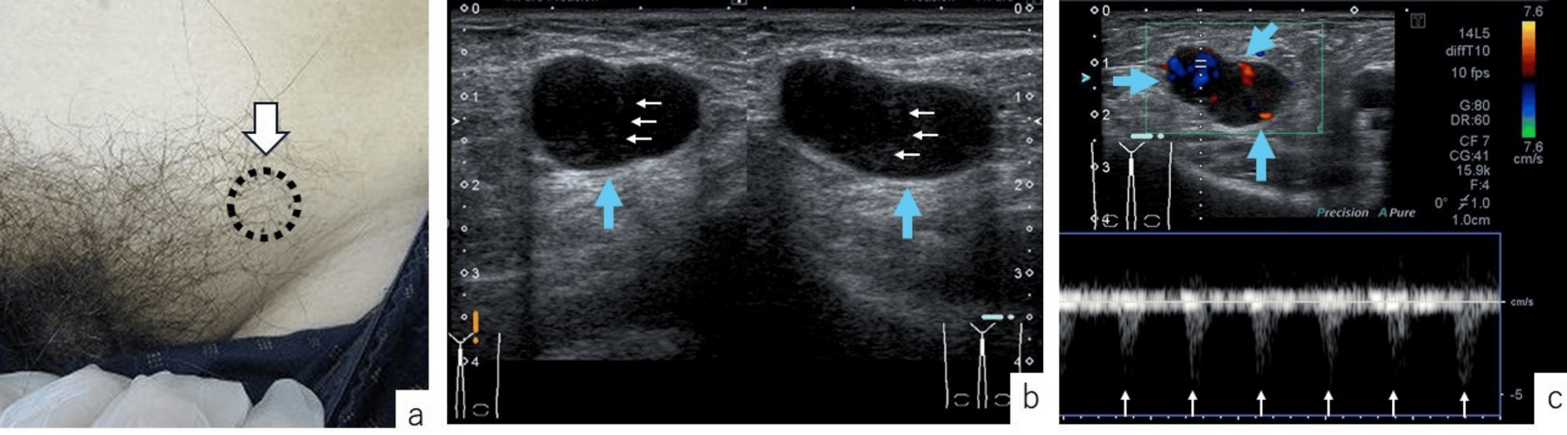
Preoperative Findings a. Initial examination findings: A painless, elastic, firm tumor approximately 25×15 mm in size (arrow) was palpable slightly cranial and medial to the left inguinal region. The tumor was not adherent to the skin or underlying tissue, and the overlying skin appeared normal. b. Findings from surface ultrasonography: Short- (left) and long-axis (right) views showed a hypoechoic tumor (blue arrows), approximately 26×14 mm in size, with septum-like structures within (white arrows), located in the subcutaneous fat layer. c. Doppler ultrasonography findings: Abundant blood flow from various directions was observed within the tumor (blue arrows), along with a regular pulsatile waveform (white arrows).

The patient was a 74-year-old man with a medical history of prostatic hyperplasia and type 2 diabetes mellitus. He noticed a mass in his left inguinal region one week before presenting to our department. On the initial examination, a painless, elastic, firm tumor measuring approximately 25×15 mm was palpable slightly cranial to and medial to the left inguinal region, without adhesion to the skin or underlying structures (Figure 1A). Ultrasonography revealed a 26×14 mm hypoechoic mass with septum-like structures within the adipose layer (Figure 1B) and abundant blood flow from various directions within the tumor (Figure 1C).

**Figure 2:**
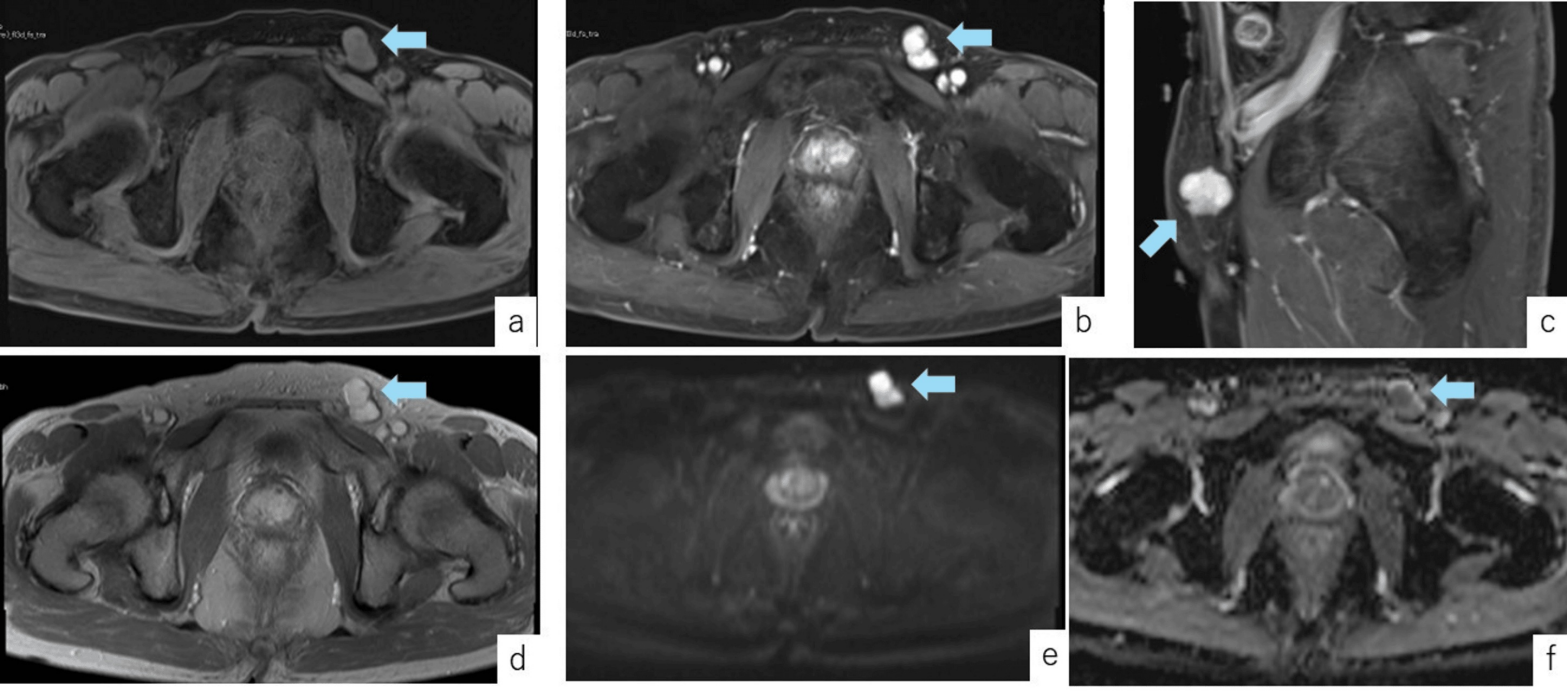
Preoperative Findings on Contrast-Enhanced MRI Contrast-enhanced MRI revealed a subcutaneous tumor in the left inguinal region, approximately 20×13 mm in size. The dynamic contrast-enhanced fat-suppressed T1-weighted sequence at 90 seconds showed heterogeneous enhancement suggestive of a lymph node tumor. Additionally, slight enlargement of the prostate and posterior bladder wall suggested the possibility of metastatic lymph node involvement. a. Fat-suppressed T1-weighted MRI (Pre-contrast, Axial View): The tumor (arrow) was located within the soft tissue. b. Fat-suppressed T1-weighted MRI (Post-contrast, Dynamic, 90 seconds, Axial View): The tumor (arrow) showed lobulated enhancement. c. Fat-suppressed T1-weighted MRI (Post-contrast, Dynamic, 90 seconds, Sagittal View): The tumor (arrow) was located below the superficial fascia. d. T2-weighted MRI Image: The tumor (arrow) was suspected to be a tumor as there was enlargement of the prostate and bladder. e. Diffusion-Weighted MRI : The tumor (arrow) showed high signal intensity. f. MRI ADC Map: The tumor (arrow) showed low signal intensity. MRI: magnetic resonance imaging, ADC: apparent diffusion coefficient

Based on these findings, lymph node swelling was suspected, and contrast-enhanced magnetic resonance imaging (MRI) was performed for further evaluation, which revealed a 20×13 mm subcutaneous mass in the left inguinal region with heterogeneous enhancement on fat-suppressed T1-weighted sequences obtained 90 seconds after contrast administration, suggestive of a lymph node tumor (Figure 2).

Additionally, slight enlargement of the prostate and posterior bladder wall, suggestive of a tumor, was observed. Based on these findings, the mass was considered likely to be a metastatic lymph node tumor. The laboratory findings are shown in Table 1. No significant findings suggestive of urological malignancies or malignant lymphoma were detected by the blood test. 

**Table 1:** Laboratory findings No significant findings suggestive of urological malignancies or malignant lymphoma were detected by the blood test.

Blood test items	Test results	Reference range
white blood cell count	5100/μL	4000-9000/μL
lactate dehydrogenase	160 U/L	124-222 U/L
C-reactive protein	0.01 mg/dL	<0.30 mg/dL
soluble interleukin-2 receptor	259 U/mL	124-466 U/mL

However, surface ultrasonographic and MRI findings raised the possibility of left inguinal lymph node metastasis from prostate or bladder cancer. Further urological investigation revealed no significant findings suggestive of prostate cancer, and cystoscopy ruled out bladder cancer. Thus, the preoperative diagnosis of the left inguinal mass was ambiguous. The patient expressed a desire for tumor resection and knowing the definitive diagnosis, leading to surgical excision and histopathological examination.

Surgical findings

Surgery was performed under local anesthesia. A transverse incision slightly longer than the tumor diameter was made along the wrinkle line directly above the tumor. The tumor was located in the adipose layer beneath the superficial fascia and was easily identified. The tumor surface appeared whitish to pale yellow with a capsule and showed no significant adhesion to the surrounding tissues. It was continuous with cord-like structures, consistent with lymph node features. The tumor was carefully dissected and excised in a manner similar to lymph node dissection. No findings suggestive of a smooth muscle tumor were observed. No other masses were identified in the surgical field.

Pathological findings 

**Figure 3:**
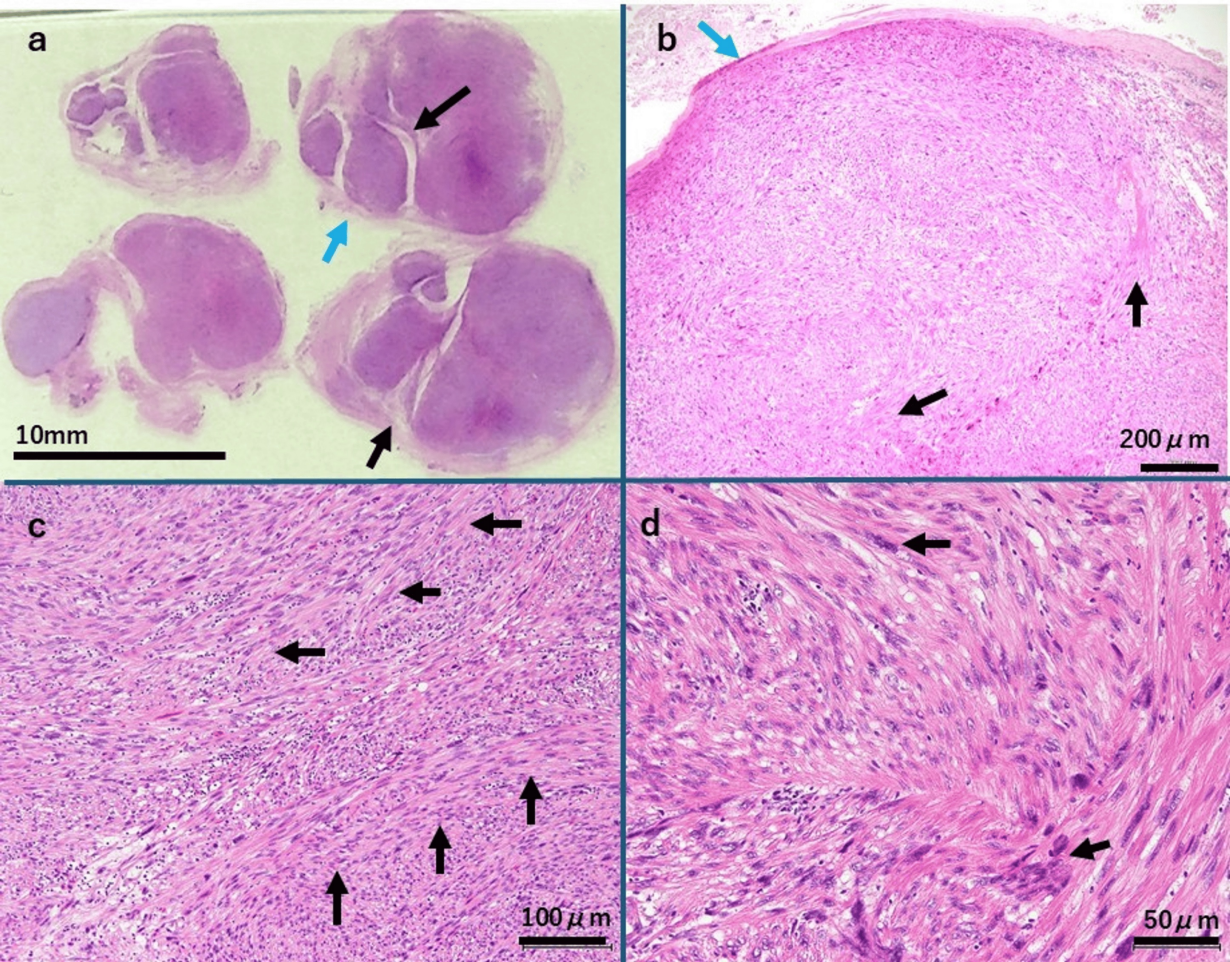
Histopathological Findings a. Low-magnification view of H&E staining: This panel presents the gross findings of the specimen on the glass slide.　The tumor was encapsulated (blue arrow), with a lobulated cross-sectional structure (black arrows). Bar: 10 mm b. H&E staining (40×): The tumor was surrounded by a capsule (blue arrow). Hyalinization was observed internally(black-arrows), but no necrotic areas were present. Bar: 200 μm c. H&E staining (100×): Spindle-shaped cells predominantly exhibited loose fascicular or interwoven arrangements (black arrows), indicating proliferation. Bar: 100 μm d. H&E staining (200×): Spindle-shaped cells displayed nuclear atypia and increased mitotic activity (black arrows). Bar: 50 μm H&E: hematoxylin and eosin

Hematoxylin and eosin (H&E) staining revealed a fibrous capsule surrounding the tumor (Figure 3A, 3B) as well as proliferating spindle-shaped cells with atypical nuclei arranged in loose bundles and interwoven patterns within the tumor (Figure 3C, 3D). The surgical margins were negative.

**Figure 4:**
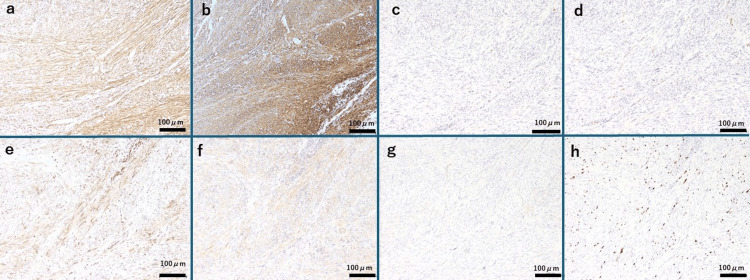
Immunohistochemical Findings a. Desmin staining: The tumor showed overall positivity, suggesting the presence of smooth muscle cells. Bar: 200 μm b. Alpha smooth muscle actin staining: The tumor was diffusely positive, suggesting the presence of smooth muscle cels. Bar: 200 μm c. S-100 Protein Staining: Negative, ruling out a neural origin of the tumor. Bar: 200 μm d. CD34 staining: Negative, ruling out a vascular endothelial origin. Bar: 200 μm e. bcl-2 staining: Faintly positive, which was inconsistent with lymphocyte or undifferentiated cell tumors. Bar: 200 μm f. STAT6 staining: Almost negative, ruling out solitary fibrous tumors. Bar: 200 μm g. β-catenin staining: Negative, excluding desmoid-type fibromatosis. Bar: 200 μm h. Ki-67 staining: Overall, 20% of cells showed positivity. Bar: 200 μm bcl-2: B cell lymphoma 2, STAT6: and signal transducer and activator of transcription 6

Immunohistochemistry (Figure 4) showed positive staining for desmin (a marker of structural proteins of muscle cells) (Figure 4A) and alpha smooth muscle actin (α-SMA, a marker of smooth muscle cells) (Figure 4B), and negative staining for S-100 protein (a marker of neural cells) (Figure 4C) and CD34 (a marker of vascular endothelial cells) (Figure 4D). Staining for B-cell lymphoma 2 (bcl-2, a marker of lymphocytes/undifferentiated cells) (Figure 4E) and signal transducer and activator of transcription 6 (STAT6, commonly expressed in connective tissue tumors) (Figure 4F) were weakly positive. These findings supported the diagnosis of ST. Additionally, staining for β-catenin (Wnt signaling pathway abnormality) (Figure 4G) and P53 were negative, and 20% positive for Ki-67 (cell proliferation marker) (Figure 4H). The H&E-stained specimen exhibited nuclear atypia (Figure 3D), increased mitotic activity, and hyalinization but no tumor necrosis or invasive features at the margins (Figure 3B, 3D), leading to the diagnosis of LM with bizarre nuclei. Following the revision of the WHO Classification of Bone and Soft Tissue Tumors to the 5th edition, which occurred soon after surgery, the pathological findings were reassessed, and the diagnosis was revised to STUMP. 

Postoperative course

The postoperative course was favorable. The tumor was initially diagnosed as a LM, with no complications, and postoperative follow-up was temporarily concluded. The WHO Classification of Bone and Soft Tissue Tumors was revised soon after surgery, and the histopathological findings were revised. We subsequently resumed follow-up for the patient after the diagnosis of STUMP, since 1) pathological findings suggestive of malignancy and excluding malignancy were mixed, 2) the criteria for malignancy in ST were not clearly defined in the revised classification, and 3) STUMP was assigned to the moderate malignancy category in the revised version. Currently, the patient is under observation, and no tumor recurrence has been observed up to the second postoperative year. We continue to monitor this patient closely.

## Discussion

Definition of STUMP

STUMP is defined as an ST of uncertain malignant potential that does not meet the diagnostic criteria for typical LM, its various subtypes, or leiomyosarcoma. Histologically, LM is characterized by intersecting fascicles of benign smooth muscle, expression of SMA and desmin, absence of necrosis, no significant nuclear pleomorphism and no/rare mitotic activity [1,2]. The latest (5th) edition of the WHO Classification introduced STUMP under the ST category. While the cause of ST is unknown [1], some reports suggest that it originates from mesenchymal stromal cells with smooth muscle differentiation potential [14]. The WHO Classification of Bone and Soft Tissue Tumors is periodically updated with the latest findings. In the 5th edition (2020) revision, knowledge gained over eight years since the publication of the 4th edition regarding molecular biology and diagnostic criteria was incorporated. In addition to STUMP, “Epstein-Barr virus-associated smooth muscle tumor” and “inflammatory leiomyosarcoma” were newly added to the ST category [1-3]. Although the criteria for malignancy of ST are controversial and vary by anatomic site and sex, the WHO Classification (5th edition) placed STUMP in the intermediate-grade tumor category [3]. In the field of gynecology, STUMP is recognized as a uterine tumor rather than a soft tissue tumor, and controversies persist around ST occurring in the uterus [4,15]. According to the General Rules for Clinical and Pathological Management of Uterine Cervical Cancer (Pathological edition), STUMP is considered an ST of uncertain malignant potential that does not meet the diagnostic criteria for typical LM, its various subtypes, or leiomyosarcoma [15]. The histopathologic characteristics of STUMP include no more than two nuclear fissions per mm2, although some cases do not meet this criterion, and there is no clear uniform indicator of malignancy [15]. In the field of gynecology, there is no consensual view on the grade of STUMP or prognostic factors.

STUMP of the uterus is also rare but is thought to occur during reproductive age and after menopause [15]. STUMP in the fields of plastic and orthopedic surgery is even rarer, with virtually no case reports or accumulated data, and its epidemiology and long-term prognosis are unknown.

About this case

The key points in the differential diagnosis of soft tissue tumors are the symptoms, clinical and imaging findings, and medical history [1]. Imaging findings in particular contribute to preoperative diagnosis, and diagnostic algorithms based on clinic-radiological features have been reported [16]. However, the preoperative diagnosis of soft tissue tumors without specific findings is difficult in many cases and is often confirmed by histopathological diagnosis. In the present case, a metastatic lymph node tumor was strongly suspected based on the presence of a mobile, painless mass in the inguinal region, history of prostatic hypertrophy, surface ultrasonography findings, MRI findings of possible prostate and bladder cancer, and MRI findings of the tumor itself. In contrast, soft tissue ST, which is rare, did not come up in the differential diagnosis. In women, STs originating from the circular ligament (attached to the uterus and extending via the inguinal canal to the subcutaneous labia majora) have been reported to occur in the inguinal region [17]. Thus, the existence of ST arising from soft tissue needs to be recognized. In addition, even in the presence of a strong suspicion of a tumor preoperatively, histopathological examination is essential for a definitive diagnosis. The histological features of neoplastic smooth muscle cells include bundle-like arrangement of spindle-shaped cells, large eosinophilic cell bodies, and long oval nuclei [3,18]. The findings of the present case were consistent with this, and therefore, the tumor was histopathologically classified as an ST. Immunohistochemical finding (positive for desmin and α-SMA, negative for S-100 protein and CD34) were also consistent with ST. In this case, the grade of malignancy was carefully determined due to the strong nuclear atypia and increased mitotic activity. The absence of tumor coagulation necrosis and increased invasion in the margin led to the diagnosis of STUMP. Even in ST of the uterus, where diagnostics are relatively advanced, STUMP has only been reported in a few cases, albeit without consensus [4,15]. STUMP, characterized by ambiguous histological features, presents significant diagnostic challenges. In 1994, Bell et al. identified three key prognostic parameters, collectively referred to as the “Stanford parameters”: a high mitotic index (≥10 mitoses/10 HPF), significant atypia (at least moderate cytologic atypia), and coagulative tumor cell necrosis. They concluded that the presence of at least two of these parameters indicates malignancy [4]. The Stanford parameters for this case are presented in Table 2.

**Table 2:** The Stanford parameters for this case In 1994, Bell et al. identified three key prognostic parameters [4]. In this case, the grade of malignancy was carefully determined due to the strong nuclear atypia and increased mitotic activity. The absence of tumor coagulation necrosis and increased invasion in the margin led to the diagnosis of smooth muscle tumor of uncertain malignant potential (STUMP).

Stanford parameters	This Case
high mitotic index (i.e., ≥ 10 mitoses/ 10HPF)	(+)
significant atypia (i.e., at least moderate cytologic atypia)	(+)
coagulative tumor cell necro sis	(-)

We inferred that the diagnosis of ST may not always align with the diagnostic criteria and that it may be difficult to determine the grade of malignancy, necessitating careful follow-up. The diagnostic criteria for STUMP remain a subject of ongoing debate, and definitive parameters for malignancy have yet to be established. Therefore, close collaboration with pathologists is essential to ensure accurate diagnosis. Furthermore, clinicians must implement careful long-term monitoring based on the diagnostic assessment. Recurrence and malignant transformation have been reported in STUMP, with prior studies recommending a postoperative follow-up period of five to 10 years [15]. Given the rarity of this case and the absence of relevant reference reports, we plan to conduct follow-up for approximately 10 years.

After surgery, the WHO Classification of Bone and Soft Tissue Tumors was revised, and the category of STUMP was added to ST. Therefore, the histopathology results of this case were reviewed. As a result, the diagnosis of STUMP was made, and the patient's follow-up was resumed. The WHO Classification is revised according to the latest findings [1,13]. Clinicians should be aware of updates and the latest information in order to optimize the treatment of soft tissue tumors. Collaboration between clinicians and pathologists is essential in the management of soft tissue tumors, as some diagnostic criteria and classifications present challenges. Our findings highlighted that closer collaboration between the two disciplines will be necessary when classifications and conventions are revised.

## Conclusions

We report a rare case of STUMP originating in the inguinal fat layer in an elderly male patient. Preoperatively, the tumor was suspected to be a metastatic lymph node tumor from a urological malignancy. Histopathological findings initially led to the diagnosis of LM with bizarre nuclei; however, reassessment according to the 5th edition of the WHO Classification led to the diagnosis of STUMP. This case highlights the necessity of pathological and immunohistochemical evaluation for diagnosing soft tissue tumors.

This case was diagnosed as an ST by histopathology and immunohistochemistry. Furthermore, the diagnosis of STUMP was made because of the high frequency of mitotic activity in the absence of coagulative necrosis in the tumor and increased infiltration at the margin. STUMP is extremely rare in the field of plastic and orthopedic surgery. Given its rarity, accurate diagnosis is crucial. For this, collaboration between clinicians and pathologists is essential, and accumulating additional case reports is also important for advancing understanding.

In view of the fact that it may be difficult to determine the grade of malignancy in STs, of which there are few reported cases and opinions are not unified, collaboration between clinicians and pathologists and careful patient follow-up are necessary.
